# Pharmaceutical services for endemic situations in the Brazilian Amazon: organization of services and prescribing practices for *Plasmodium vivax *and *Plasmodium falciparum *non-complicated malaria in high-risk municipalities

**DOI:** 10.1186/1475-2875-10-335

**Published:** 2011-11-03

**Authors:** Martha C Suárez-Mutis, Paula P de Souza, Letícia F Freitas, Elaine S Miranda, Mônica R Campos, Claudia GS Osorio-de-Castro

**Affiliations:** 1 Laboratório de Doenças Parasitárias, Instituto Oswaldo Cruz Fundação Oswaldo Cruz, Av. Brasil 4365, Pavilhão Arthur Neiva, sala 21-B, CEP - 21040-900, Rio de Janeiro, Brazil; 2 Núcleo de Assistência Farmacêutica, Escola Nacional de Saúde Pública Sérgio Arouca, Fundação Oswaldo Cruz, Rio de Janeiro, Brazil; 3 Departamento de Farmácia e Administração Farmacêutica, Faculdade de Farmácia, Universidade Federal Fluminense, Rio de Janeiro, Brazil; 4 Departamento de Ciências Sociais, Escola Nacional de Saúde Pública Sérgio Arouca, Fundação Oswaldo Cruz, Rio de Janeiro, Brazil

## Abstract

**Background:**

In spite of the fact that pharmaceutical services are an essential component of all malaria programmes, quality of these services has been little explored in the literature. This study presents the first results of the application of an evaluation model of pharmaceutical services in high-risk municipalities of the Amazon region, focusing on indicators regarding organization of services and prescribing according to national guidelines.

**Methods:**

A theoretical framework of pharmaceutical services for non-complicated malaria was built based on the Rapid Evaluation Method (WHO). The framework included organization of services and prescribing, among other activities. The study was carried out in 15 primary health facilities in six high-risk municipalities of the Brazilian Amazon. Malaria individuals ≥ 15 years old were approached and data was collected using specific instruments. Data was checked by independent reviewers and fed to a data bank through double-entry. Descriptive variables were analyzed.

**Results:**

A copy of the official treatment guideline was found in 80% of the facilities; 67% presented an environment for receiving and prescribing patients. Re-supply of stocks followed a different timeline; no facilities adhered to forecasting methods for stock management. No shortages or expired anti-malarials were observed, but overstock was a common finding. On 86.7% of facilities, the average of good storage practices was 48%. Time between diagnosis and treatment was zero days. Of 601 patients interviewed, 453 were diagnosed for *Plasmodium vivax*; of these, 99.3% received indications for the first-line scheme. Different therapeutic schemes were given to *Plasmodium falciparum *patients. Twenty-eight (4.6%) out of 601 were prescribed regimens not listed in the national guideline. Only 5.7% individuals received a prescription or a written instruction of any kind.

**Conclusions:**

The results show that while diagnostic procedure is well established and functioning in the Brazilian malaria programme, prescribing is still an activity that is actually not performed. The absence of physicians and poor integration between malaria services and primary health services make for the lack of a prescription or written instruction for malaria patients throughout the Brazilian Amazon. This fact may lead to a great number of problems in rational use and in adherence to medication.

## Background

Brazil today reports 50% of all malaria cases in the Americas Region and the Amazon accounts for 99.8% of all cases in the country [1]. Over the years, fluctuations in the number of malaria cases have occurred. However, social changes and internal migration brought an upsurge of malaria [2]. In 2005, there were more than 600, 000 reported malaria cases. With firm implementation of control measures, by the Brazilian National Malaria Control Programme (PNCM), in 2009 the number of notifications dropped to 308, 000, reaching the specific 2010 Millennium Goal for malaria in the country [3].

The PNCM's adopted control rationale focuses on early diagnosis and adequate treatment, while also favouring prevention strategies. All malaria actions are under the responsibility of the Brazilian government. After 2003, the federal control of malaria activities was transferred to state and local (municipal) control. A network of malaria outposts in the Amazon region provides free microscopy diagnosis, treatment with standard medicines and selective vector control [4]. Lately, health education and delivery of long-lasting impregnated bed nets was incorporated to control strategies. By 2008, there were 3422 laboratories for microscopy diagnosis and 48, 281 primary health care agents for malaria care in 807 municipalities; in that year, the number of high risk (API > 50) municipalities was 67 [5]. Almost 85% of malaria cases in the country are caused by *Plasmodium vivax*. [1]. Although the emergence of chloroquine resistance in Manaus (State of Amazonas) has been reported [6], the treatment for *P. vivax *is still centered on the combination of chloroquine and primaquine.

Treatment for *Plasmodium falciparum *in Brazil had traditionally involved quinine sulphate, doxycycline and primaquine. Growing resistance, complexity of regimen, adverse effects and sub-optimal therapeutic effectiveness [7] led to the introduction of an artemisinin-based combination therapy (ACT), at the end of 2006, by the PNCM in some few municipalities. At the time of the study, the first ACT combination was introduced, artemether-lumefantrine. With these new combinations, the proportion of *P. falciparum *malaria cases decreased from 31% in 2002 to 15% 2009 [8].

The therapeutic use of anti-malarials may lead to non-adherence, enhancing resistance and disease prevalence. In this topic, quality pharmaceutical services are considered a key element for control, especially considering the PNCM's rationale [9].

In spite of the fact that pharmaceutical services are an essential component of all malaria programmes, especially those that focus on early diagnosis and adequate treatment, quality of pharmaceutical services for malaria has been little explored in the literature. The most published information concerns therapeutic regimens for *P. falciparum *[10]. In fact, it is the need for quality services, rather than the species, which should be the central point of the argument [11].

**Figure 1:**
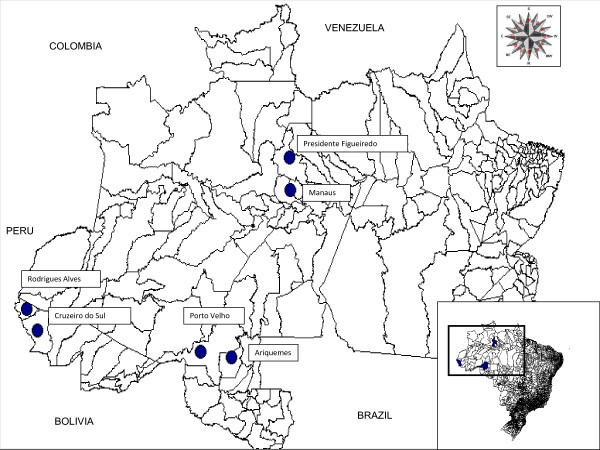
**Map of selected municipalities in the Brazilian Amazon**. Six malaria high risk municipalities in the Brazilian Amazon were selected for the study. Cruzeiro do Sul and Rodrigues Alves in the State of Acre, Manaus and Presidente Figueiredo in Amazonas and Porto Velho and Ariquemes in the State of Rondonia. The election criteria were API (Annual Parasitic Index) greater than 50 cases per 1, 000 inhabitants (in the previous year) and an ocurrence of at least 7, 000 cases of malaria per year (in order to guarantee 150 patients consulted per week). Finally, two municipalities with > 100, 000 inhabitants (Manaus and Porto Velho), 2 with between 99, 999 and 30, 000 inhabitants (Ariquemes and Cruzeiro do Sul) and 2 with < 29, 999 inhabitants (Presidente Figueiredo and Rodrigues Alves) were selected from eligible municipalities in the region.

In order to understand comprehensive pharmaceutical services (such as the organization of services, prescribing and dispensing) for malaria and other endemic diseases, a theoretical outline composed of a logical model and a framework was proposed as an evaluation model of these services [12]. This study presents the first results of the application of this evaluation model in six high-risk municipalities of the Amazon region (Figure 1), focusing on organization of services and prescribing according to national guidelines.

## Methods

The methodology is based on the theoretical framework of the "Pharmaceutical services for non-complicated malaria by *P. vivax *and *P. falciparum *in high-risk municipalities of the Brazilian Amazon: organization of services, prescribing, dispensing and adherence to treatment" The Mafalda Project, published previously [12]. A broad review of pharmaceutical services for malaria was undertaken in the literature, from 1980 to 2005 [10]. An analytical outline, comprising the theoretical model and 25 indicators was developed. These indicators included the following dimensions: context and organization of pharmaceutical services, prescribing, dispensing and adherence to treatment. This paper will focus on the first two dimensions. Dispensing and adherence will be developed in a forthcoming paper.

The investigative strategy was based on the Rapid Evaluation Method, proposed by the World Health Organization (WHO) [13] and adapted by Management Sciences for Health (MSH) [14,15]. In evaluations of non-complicated malaria a sample of not under 600 patient registries is recommended [16].

### Study sites and sample

In Brazil, malaria control is based on early diagnosis and treatment, which are entirely carried out by the National Malaria Control Programme (PNCM). All healthcare for malaria is universal and free and all facilities must adopt the National Treatment Protocol (MTM). All treatment regimens investigated in this paper followed the version adopted at the time of the study [17].

The study was carried out in six malaria high-risk municipalities of the Brazilian Amazon. The municipalities were first selected according to API (Annual Parasitic Index) greater than 50 cases per 1, 000 inhabitants, in 2005 and a count of, at least, 7, 000 cases of malaria per year (in order to guarantee 150 patients consulted per week in each municipality). Once these criteria were met by all the high-risk municipalities, they were further examined for the adoption of the 2001 national guideline (MTM) and the admission, by local health managers, that a prescription or a written instruction was given to the malaria patient. These criteria determined 15 eligible municipalities in 4 states. The following step included the stratification according to population size, in order to cover possible differences in services structure. Therefore, two municipalities with > 100, 000 inhabitants, two with 99, 999 to 30, 000 inhabitants and two with < 29, 999 inhabitants were selected from the eligible municipalities. The final choice was based on proximity, in order to make field logistics in the Amazon easier.

Eligible health units were all primary health facilities and health centers in municipal urban areas involved in Primary Health Care (PHC) and distancing not more than 50 km from city-center. In spite of the fact that two municipalities (Manaus and Porto Velho) harbored reference centers for malaria, none were visited in this part of the investigation. Health teams for malaria care were composed mainly by health agents. Their training and experience is described elsewhere [18]. Up to four health facilities were visited in each municipality in order to attain the required number of patients per municipality. The number of visited health facilities in each municipality was not standardized since the chosen unit of analysis was the municipality and not the health unit. Organization of services focused on pharmaceutical services in primary health care facilities and not on all levels of health care in the municipality.

The investigation was limited to individuals of both genders ≥ 15 years of age. Patients presenting with symptoms confirmed by positive thick smear were followed throughout prescribing and dispensing.

### Data collection instruments

All data collection instruments (questionnaires, observation forms, interviews forms and charts review forms) were prepared according to the theoretical framework [12] and applied successively during project data collection. Interviews were conducted based on objective questionnaires and interview forms for qualitative data. Two different observation forms were used to collect service data (organization of pharmaceutical services, stock and management of anti-malarials) and to organize recruitment of individuals. A chart review form compiled data on patient diagnoses, type of *Plasmodium spp *and time from diagnosis to treatment. An additional chart review form examined prescription data (compliance to national guideline). Direct observation was also undertaken by field researchers for examination of stock management and of pharmaceutical services routines.

### Pilot study

A pilot study was conducted in a high-risk municipality not on the study sample (Tucuruí, Pará), in order to apply and adjust the methodology and the data collection instruments.

### Field study

Research teams were trained on project objectives and on data collection procedures and instruments in order to increase internal consistency and reliability of data collection. Two teams were deployed, each visiting three different municipalities. Data collection took approximately 10 days for each municipality. Previous contact was established with all malaria managers and written consent was given before visits. Researchers were instructed to start by visiting the local health service to engage in a person-to-person explanation of the study and to broker an overall receptive environment for the investigation.

Investigation started with organization of pharmaceutical services in municipalities and in health facilities. Malaria consultation days were determined for optimal patient recruitment. Before recruitment, patients were asked to participate and consent was given through a written statement.

### Data analysis and statistical procedures

Data was checked by independent reviewers and fed to a data bank (built in Access for Windows^®^, Microsoft Corp., 2007) through double-entry. Inconsistencies were verified and corrected. Data was descriptively analysed and tabulation was used to organize main results.

### Ethical considerations

This study was approved by the Ethics in Reasearch Committe of the Sergio Arouca National School of Public Health, Oswaldo Cruz Foundation (Fiocruz). (Approval number 91/06; CAAE 0086.0.031.000.06)

## Results

### Context and pharmaceutical services organization

The 2007 API classified the studied municipalities as high malaria risk, except for Manaus (AM) and Ariquemes (RO), which had been high-risk in 2005. However, malaria distribution was not homogeneous, varying from 28.7/1000 inhabitants in Manaus to 572.8/1000 inhabitants in Rodrigues Alves. In 2008, there was a decrease in the number of malaria cases in all the municipalities, but Rodrigues Alves (AC), Cruzeiro do Sul (AC) and Presidente Figueiredo (AM) were still stratified as high-risk malaria areas. In all municipalities, *P. vivax *was the most prevalent *Plasmodium *both in 2007 and in 2008 (Table 1).

**Table 1:** Distribution of malaria cases in 2007 and 2008 in selected municipalities of the Brazilian Amazon

		2007				2008			
**Municipality**	**Population**	**Cases**	**API**	***% vivax***	***% falciparum***	**Cases**	**API**	***% vivax***	***% falciparum***

Rodrigues Alves, AC	13.460	7.710	572, 8	81, 2	19, 2	3.118	231, 6	82, 2	17, 8

Cruzeiro do Sul, AC	77.004	28.339	368, 0	80, 5	19, 5	15.407	200, 1	83, 0	17, 0

Manaus, AM	1.738.641	49836	28, 7	88, 9	11, 7	24.974	14, 4	86, 2	13, 8

Presidente Figueiredo, AM	26.282	4.311	164, 0	86, 0	14, 4	3.235	123, 1	89, 5	10, 5

Ariquemes, RO	85.541	3.618	42, 3	72, 0	28, 0	2.194	25, 6	79, 7	20, 3

Porto Velho, RO	382.829	32.934	86, 0	78, 9	21, 1	23.648	61, 8	85, 4	14, 6

API: Annual Parasitic Index

Source: Virtual Health Library. Ministry of Health. Brazil.

Pharmaceutical services organization were evaluated according to indicators stemming from the "Mafalda" logical model [12]. Fifteen facilities were investigated in six municipalities (Ariquemes, Cruzeiro do Sul, Manaus, Porto Velho, Presidente Figueiredo and Rodrigues Alves (Figure 1). Of these, 12 (80%) presented a copy of the official guideline for malaria treatment (*Manual de Terapêutica da Malária *- MTM). Availability of other, additional instruction material, such as pamphlets produced by the PNCM or by the state health service, in order to offer information on the recently introduced anti-malarial treatment with ACT, was detected in 13 of 15 (or 87%) facilities. Ten out of 15 (67%) facilities presented an environment for receiving patients and for prescribing.

The availability of complete treatments for *P. vivax *and *P. falciparum *was estimated considering that primaquine, at the time, was used for both treatments in different total doses. The same amount of medicine would treat one patient for *P. vivax *and 4.6 patients with *P. falciparum*. In each municipality re-suply of stocks followed a different timeline (monthly, weekly and daily re-stock routines were observed), but it was evident that none of them adhered to forecasting methods for stock management. As a result, in spite of the fact that no shortages were observed for any anti-malarial, overstock was a common finding. In Manaus and in Porto Velho, quinine sulphate was overstocked, while Manaus, Porto Velho and Ariquemes presented overstock of doxycycline. Chloroquine and primaquine were overstocked in Presidente Figueiredo, considering regimens for both species.

Adequate storage practices were evaluated using a 10-item checklist (Table 2). Only two of the 15 facilities had separate storage and dispensing areas. These two storage areas scored only 70% of good practices while the corresponding dispensing areas scored 90% and 60%. All other 13 facilities presented medicine stocks only in dispensing areas. In these, the average good storage practice score was 48%. Porto Velho scored worst among all municipalities, because facilities presented a meager 30% average in good storage practices.

**Table 2:** 10-point checklist for evaluating storage practices for anti-malarials

NUMBER	ITEMS
1	Space and surface areas are clean and free of dust.
2	Space (roofs, floors, countertops and walls) present adequate conditions for cleanliness and orderliness (light-colored, washable, easy-to-clean) and for maintenance (moisture-free, water-protected, properly sealed).
3	Space provides proper air circulation (e.g. ceiling space at least 3 meters high, vents, exhaust fans etc.)
4	Space provides temperature-control (e.g., air-conditioning).
5	Products are protected from direct sunlight (e.g. windows are painted, curtains or shades are provided).
6	Medicines are stored with no contact with floor or walls (for which shelves, pallets etc are provided.).
7	Medicines are stored methodically (e.g., alphabetically, by therapeutic class, by expiry date etc).
8	Space is free of pests (insects, birds, rodents, marsupials etc.).
9	Fire extinguishers are operational, with valid expiry date and access to them is clear.
10	There is an inventory control system

No expired anti-malarials were found, in any facility. All 601 reviewed treatments were indicated by health staff and dispensed after laboratory results, but in 24 (4%) cases species was not determined and treatment was empirical. Average time between diagnosis and treatment was zero days, *i.e*., thick smear was made immediately on arrival at the health facility. Of laboratory-diagnosed patients in which species was identified, 453 were diagnosed for *P. vivax*, 120 for *P. falciparum*, two for mixed malaria (*P. vivax *+ *P. falciparum*), two other species and 24 were not identified.

Of 63 health workers and health professionals involved in malaria treatment at the 15 facilities, only one was a medical doctor, stationed in Presidente Figueiredo. All professionals and workers presented some level of training for malaria care, but the level of knowledge and quality of practice varied, reflecting upon their perceptions as to diagnosis, treatment regimens, interaction with patients, and responsibilities [18].

### Prescribing and adherence to national guidelines

Prescribing practices were also evaluated according to the "Mafalda" study [12]. A total of 601 patient charts were reviewed at the 15 health facilities and when possible prescribing underwent observation. Of these, in only 34 cases (5.7%), patients received a prescription or a written instruction of any kind on how to take their medicines. In Ariquemes, Cruzeiro do Sul, Porto Velho and Rodrigues Alves none of the patients received prescriptions. In Manaus 20/128 (15, 6%) individuals received written instructions and in Presidente Figueiredo, the only municipality where a doctor attended to malaria patients, prescriptions were issued to 14 in 91 patients (15, 4%).

In relation to treatment indications according to national guidelines, of the 453 (75.4%) patients diagnosed for *P. vivax *malaria, indications for first-line scheme (chloroquine and primaquine) were passed to 450 (99.3%) patients. Of the 120 patients (20.0%) diagnosed with *P. falciparum *malaria, first-line treatment (quinine-doxycycline-primaquine) was indicated to 57 (47.5%) patients. Three (2.5%) patients were medicated with mefloquine and primaquine (second-line treatment scheme) and 26 (21.7%) with lumefantrine-artemether. Of the 34 patients that received indication for other treatments not officially sanctioned by the Ministry of Health, 27 (22.5%) were medicated with a new ACT combination (mefloquine-artesunate) under study by the PNCM at the time, in Cruzeiro do Sul (AC). No information was available on treatment indicated for seven (5.8%) patients. Of recommended anti-malarials, dosage forms and concentrations followed MTM 2001 specifications. Twenty-eight (4.6%) out of 601 were prescribed either for regimens for other types of malaria or empirically for regimens not listed in the national guideline.

## Discussion

Malaria, as a public health priority, is an important focus point for development of quality pharmaceutical services, and can serve as a model for other endemic diseases in tropical areas of the world. This study encompassed a special period for the PNCM, since the 2001 guideline was being reviewed and new ACT schemes were being introduced for *P. falciparum*.

The assumptions in studying pharmaceutical services in high-risk municipalities were simple. First, the Brazilian control strategy is based on diagnosis and treatment, making pharmaceutical services essential. Second, pharmaceutical services must be organized in those endemic diseases areas where health needs are critical. Third, it is important to study highly endemic areas, as services are apt to be more structured and organized that in others where prevalence is low and the disease is not perceived as a health risk. Finally, the choice of municipalities was intentionally made to include not only incidence, but different population magnitude, to reflect municipal diversity in the Brazilian Amazon.

Although choice of municipalities may be perceived as a limitation of this study and may have implications on generalization of results, especially for rural areas, the results may be applicable to urban areas in the Brazilian Amazon. Furthermore, the effort was made to give a comprehensive view of organization of services and of prescribing in municipalities of various population sizes once it was estimated that population size was a good predictor for services organization [12].

Results showed that *P. vivax *malaria is the most prevalent species found in the visited municipalities, repeating the pattern observed in the Brazilian Amazon [5]. Manaus and Ariquemes, which had been high-risk municipalities in 2005, were excluded from this category in 2007 (Table 1). Number of cases dropped considerably in all municipalities in 2008. However, only these two maintained their high-risk-free status.

It was observed that 80% (12/15) of facilities had an available copy of the 2001 official guideline for malaria treatment and this is a positive indicator of quality of care. Nevertheless the guideline itself had problems, since it was based on limited pharmaceutical and clinical evidence [10]. In 2010, the PNCM published new guidelines with better quality and based on more consistent and locally-generated scientific evidence [8].

Additionally, 87% of health services had other instructional material. However this information had not been disseminated to all the health workers in the area [18]. An especially critical issue in rational medicines use is availability of information, for users and for health professionals alike. Uninformed health professionals may be unable to treat patients adequately. Reports show that with appropriate information even patients with little education were able to follow treatment with artemether-lumefantrine, without supervision [19,20].

Another positive aspect was that no expired anti-malarials were found. Nonetheless, overstock was detected and none of the municipalities adhered to forecasting methods of stock management. None of the health facilities fulfilled 100% of good practices in storage and dispensing areas. As was to be expected, re-supply of stocks followed no discernible routines, in spite of a perceived sense of urgency in making treatment available. Forecasting activities are an essential part of pharmaceutical services organization. This aspect is tantamount, especially in the Amazon, where humidity and temperatures are very high most part of the year. In fact, a sampling of anti-malarials from Brazilian treatment facilities was submitted to quantitative analytical assays and presented problems in concentration of the active principles [21]. The evidence suggests that if in urban areas this situation was detected, in rural regions, where facilities may be less than adequate, with deficiencies in storage and dispensing areas, where staff is possibly lacking and large distances may cause supply shortages, the situation should be worse, as has been cited in the literature [22,23].

In respect to diagnostic coverage, this study showed that all individuals were submitted to laboratory confirmation of malaria and that in only 24 patients (4%) species was not identified and that these were given empirical treatment. In spite of reflecting only urban areas, this operational indicator showed good performance, following WHO recommendations (at least 90% of suspected malaria outpatient cases should undergo laboratory diagnosis-thick smear or Rapid Diagnostic Test) [24]. Different results are reported from some African countries, where laboratory diagnostic coverage revolved from 33% to 40% [25,26], while in other settings 60% of patients have no access to diagnosis [27].

Maybe the failures in diagnosis were due to probable test error. Albeit considered simple and less technologically intense that other laboratory methods, the thick smear may be a tricky technique. It is prone to considerable inter-observer variation, while requiring continuous training of staff, as well as minimum laboratory infrastructure [28]. Brazil has recently introduced a new career for health workers, that of "microscopy technician", to give professional incentives to health technicians working in malaria [29], decreasing rapid turnover of staff in endemic areas and consequently favoring diagnostic quality.

The findings of this study show that patients have close to immediate access to microscopic diagnosis. The average time between diagnostic and treatment was 0 days. This was considered an excellent result, considering other examples in the literature [30]. In the state of Amazonas 57% of patients are offered treatment within 48 hours of symptoms. In Rondonia and in Acre, the proportions are 65% and 71% respectively. The national Brazilian average is 59% [31]. The findings may reflect the urban scenario depicted in the study.

Complete regimens for both *P. vivax *and *P. falciparum *were readily available in the visited facilities. For *P. vivax *patients, treatment indication according to the national guidelines was successful, while for *P. falciparum *patients were offered more alternatives. During the study period only one first-line regimen for uncomplicated malaria in adults was recommended for *P. vivax*. For *P. falciparum*, the guideline offered two alternatives, because of *Plasmodium *resistance and a third as consequence of introduction of ACTs in Amazonas.

Nonetheless, a percentage of treatments did not comply with the national guidelines. While one ACT fixed-dose combination was being introduced in Amazonas, another was undergoing clinical field study in Acre - an intervention involving 17, 000 patients and sponsored by The Brazilian Ministry of Health and by Ravreda (*Rede Amazônica de Vigilância de Resistência aos Anti-maláricos*) [4]. The tentative incorporation of ACT to the treatment guideline was, therefore, not uniform throughout the Amazon. The low number of empirical treatments was due to diagnostic failure and not to inobservance of the national protocol.

Only 5.7% of individuals with malaria received prescriptions or written information on treatment regimens. Only one medical doctor was identified among 63 health workers involved in malaria treatment in the six study sites. These facts reflect part of the malaria care-giving urban scenario in Brazil. Nonetheless, in reference centers for malaria in the Brazilian Amazon, all individuals with malaria are attended to by medical doctors [32]. Brazilian Health System and PNCM policy recommends that better clinical evaluation of each case should be guaranteed through physician-attended care. Therefore, it is difficult to accept that in the visited municipalities a lesser standard of care should be acceptable. Decentralized health interventions have been considered as advances of the Brazilian Health System, in regard to access for users and accountability for municipal managers, but differences in revenue, demographics, health indicator performance and in quality of management result in better or worse malaria control [4].

In spite of the fact that one of the inclusion criteria was the availability of a prescription or a written instruction to the patient, this was not observed in the field. The existence of a medical prescription is a very important concern since malaria should be treated with a combination of drugs, and the therapeutic scheme depends on the parasitic species, patient age group and clinical severity of the disease. Moreover, many malaria patients have little formal education [33] and the complexity of the therapeutic regimens for both *P. vivax *and *P. falciparum *may baffle them. They rely on family or friends to help with treatment schemes, making a clear readable prescription therefore important to enhance adequate adherence to a complete treatment [34,35]. The lack of a formal prescription may have, therefore, made the actual treatment regimen given to each patient difficult to detail, since the study relied mostly upon observation and chart reviews.

It is possible to hypothesize that the rest of non-ACT/non-protocol treatments for *P. falciparum *may have been caused by the lack of medical doctors and pharmacists and by deficient training of other health professionals. Trained staff could nullify or bar inadequate medicines indications. Adherence to therapeutic guidelines goes hand in hand with capacity building of health staff and available information on treatment [22,25].

Only two thirds of the visited facilities presented a specific area for receiving and consulting patients. In pharmaceutical services, a proper environment for patient dispensing and counseling is a determinant in the patient's understanding of the treatment regimen and of possible adverse effects caused by medicines. Layout of patient facilities is recognized as fundamental in guaranteeing better patient comprehension [25]. This, in turn, may lead to good or bad adherence to treatment [33].

It is worthwhile to point out that a specific programme for malaria with a separate structure of pharmaceutical services, although helping to focus and prioritize disease needs, may be more difficult to manage. In Brazil, municipalities are responsible for primary healthcare. Although legislation has been passed stressing the importance of combining efforts between primary health care (PHC) and malaria [36], joint work is absent or lacking in most municipalities. The fact that the PNCM is a vertical programme within the Ministry of Health and centrally managed in regard to procurement is not an excuse for the problems observed in pharmaceutical services. Other vertical programmes have shown a better capacity for integration with PHC [37]. Pharmaceutical services would profit by viewing malaria from the perspective of PHC since all phases, from forecasting to rational use of medicines, apply to both. The literature has observed that difficulties in malaria control are greater where integrations with PHC is lacking [38].

## Conclusions

Malaria is a public health priority. The Millennium Goals and the Roll Back malaria partnership state that, by 2015, total malaria cases have to be reduced to levels 75% lower than those of the year 2000 [39,40]. In Brazil, the reduction goal for 2010 has been achieved [5], but because malaria is such a recurrent disease, with a complex biological cycle, and strongly influenced by social and economic determinants, this result only warrants the need to strengthen basic health and malaria services.

According to Greenwood [41], malaria cannot be eliminated if quality of health systems which deliver care and treatment is not accomplished first. There has been considerable research directed to the eradication/elimination of the disease, in the form of R&D of new molecular entities active against *Plasmodium spp *or vaccines; but research on health services for malaria is equally important and noteworthy. In programmes where treatment is tantamount, pharmaceutical services research must be acknowledged as fundamental. In respect to them, few reports are available in the literature [26].

The visited facilities presented problems with organization of services - especially concerning stock management and storage. It was decided not to include rural areas, resulting in exclusion of rural health units. In spite of this limitation, in urban areas - where patients have better access to health services and more formal schooling - better facilities and services are expected. It is suggested that the organization of pharmaceutical services in rural malaria services may be worse. Additionally, the visited municipalities accounted for an important percentage of malaria cases in 2006-2007, and in them malaria is one of the main health problems. Because of this, it could be considered that malaria services and pharmaceutical services for malaria in these municipalities should have shown a higher level of organization.

These results show that while diagnostic procedure is well established and functioning in the PNCM, prescribing is still an activity that is actually not performed. The absence of physicians and the poor integration between malaria services and primary health care make for the lack of a prescription or written instruction for malaria patients in several high-risk municipalities in the Brazilian Amazon. This fact may lead to a great number of problems in rational use and in adherence to medication.

## Competing interests

There is no conflict of interest from any of the authors of the manuscript due to commercial or other affiliations.

## Authors' contributions

CGSOC and MCSM contributed to the conception and the design of the study, developed the questionnaire, collected the data, and contributed to the analysis and interpretation of the data and to the writing of the paper; MRC and ESM contributed to the conception and the design of the study, developed the questionnaire, collected the data, and contributed to the analysis and interpretation of the data; PPS and LFF contributed to the analysis and interpretation of the data and wrote the paper. All authors put forward different ideas, contributed to the interpretation of the data, early drafts and agreed the final draft.
